# Hydrothermal phase transformation of hematite to magnetite

**DOI:** 10.1186/1556-276X-9-230

**Published:** 2014-05-13

**Authors:** Jie-feng Lu, Cho-Jen Tsai

**Affiliations:** 1 Department of Material Science and Engineering, National Tsing-Hua University, Hsinchu 30013, Taiwan

**Keywords:** Iron oxides, Hydrothermal, Phase transformation

## Abstract

Different phases of iron oxide were obtained by hydrothermal treatment of ferric solution at 200°C with the addition of either KOH, ethylenediamine (EDA), or KOH and EDA into the reaction system. As usually observed, the α-Fe_2_O_3_ hexagonal plates and hexagonal bipyramids were obtained for reaction with KOH and EDA, respectively. When both KOH and EDA were added into the reaction system, we observed an interesting phase transformation from α-Fe_2_O_3_ to Fe_3_O_4_ at low-temperature hydrothermal conditions. The phase transformation involves the formation of α-Fe_2_O_3_ hexagonal plates, the dissolution of the α-Fe_2_O_3_ hexagonal plates, the reduction of Fe^3+^ to Fe^2+^, and the nucleation and growth of new Fe_3_O_4_ polyhedral particles.

## Background

The more stable phases in iron oxides are hematite and magnetite. Hematite can be used in a lot of applications, such as sensors [1], water photooxidation [2], drug delivery [3], lithium ion battery [4], pigmentation [5], solar cell [6], etc., and magnetite can be utilized in biomedicine [7-11], magnetic devices [12], etc. Therefore, studies about the nano/microstructures of iron oxides and their properties, which are related to the intrinsic structure and crystal shapes, have been intensively engaged, especially for hematite and magnetite. The bandgap of hematite is 2.0 to 2.2 eV which makes it useful in applications that involve visible light absorption [13,14]. Magnetite has unique electric and magnetic properties because its intrinsic crystal structure allows electrons to be transferred between Fe^2+^ and Fe^3+^ in the octahedral sites [15].

Many researches have demonstrated the capability of using chemical syntheses to control particle morphologies of iron oxide by surfactants [16-18]. Morphologies like wires [19], rods [20], tubes [21], rings [22], disks [23], cubes [24], spheres [25], hexagonal plates of α-Fe_2_O_3 _[26,27], and polyhedral particles of Fe_3_O_4 _[28,29] have been synthesized successfully.

Several robust methods have been developed for phase transformation of iron oxides. α-Fe_2_O_3_ can be transformed to Fe_3_O_4_ at high temperature under a reducing ambient, such as hydrogen ambient [30,31]. Yanagisawa and Yamasaki also showed that by controlling the mineralizer solutions, temperatures, and partial pressures of hydrogen in a hydrothermal system, phase transformation from α-Fe_2_O_3_ to Fe_3_O_4_ particles can be achieved [32]. The result indicated that high temperature and high pressure of hydrogen can accelerate the reduction reaction.

Phase transition of iron oxides can also take place by hydrothermal reaction with a reducing agent [33,34]. Sapieszko and Matijewic had observed a similar phase transformation from α-Fe_2_O_3_ hexagonal plates to octahedral Fe_3_O_4_ particles triggered by the addition of hydrazine which is used as an antioxidant [35] during hydrothermal process.

In this experiment, we explore the role of ethylenediamine (EDA or en in ligand form) on the phases of iron oxide in hydrothermal condition. EDA is usually considered to be the chelating agent or to function as a ligand to facilitate the growth of particles under hydrothermal reaction [36,37]. However, phase transformation of iron oxide was observed when EDA was added into the alkaline solution. Thus, a special low-temperature route for the transformation of α-Fe_2_O_3_ to Fe_3_O_4_ was provided. The phase and shape variations with the addition of potassium hydroxide (KOH), EDA, and KOH and EDA were investigated and compared.

## Methods

Ferric nitrate (Fe(NO_3_)_3_ · 9H_2_O), 1 mmol, was dissolved in 10 ml of distilled water to form a transparent yellow solution. Next, three different mineralizing agents were added into the ferric solution. First is 5 ml of 10.67 M KOH aqueous solution. The solution was added dropwisely into the ferric solution. Second is 1 ml of EDA. The EDA was added gradually into the ferric solution. Third is the combination of KOH and EDA. The 10.67 M KOH solution, 5 ml, was added first followed by the addition of 1 ml of EDA. After adding these mineralizing agents, a brown Fe(OH)_3_ suspension was obtained. Then, these solutions were all stirred for 30 min before transferring the mixture into a Teflon-lined stainless steel autoclave (DuPont, Wilmington, DE, USA) of 40-ml capacity and followed by heat treatments at 200°C for 9 h. After that, the autoclave was cooled down to room temperature in air. The precipitates were collected by centrifugation, washed with deionized water and ethanol several times to remove organic and impurities, and finally dried in air at 80°C for 12 h.

The as-synthesized powder was characterized by X-ray diffraction (XRD) with Cu-Kα radiation, field emission scanning electron microscopy (FE-SEM), transmission electron microscopy (TEM), and Raman spectroscopy. The magnetic properties were measured by a vibrating sample magnetometer (VSM) with a maximum magnetic field of 1.5 kOe.

## Results and discussion

**Figure 1:**
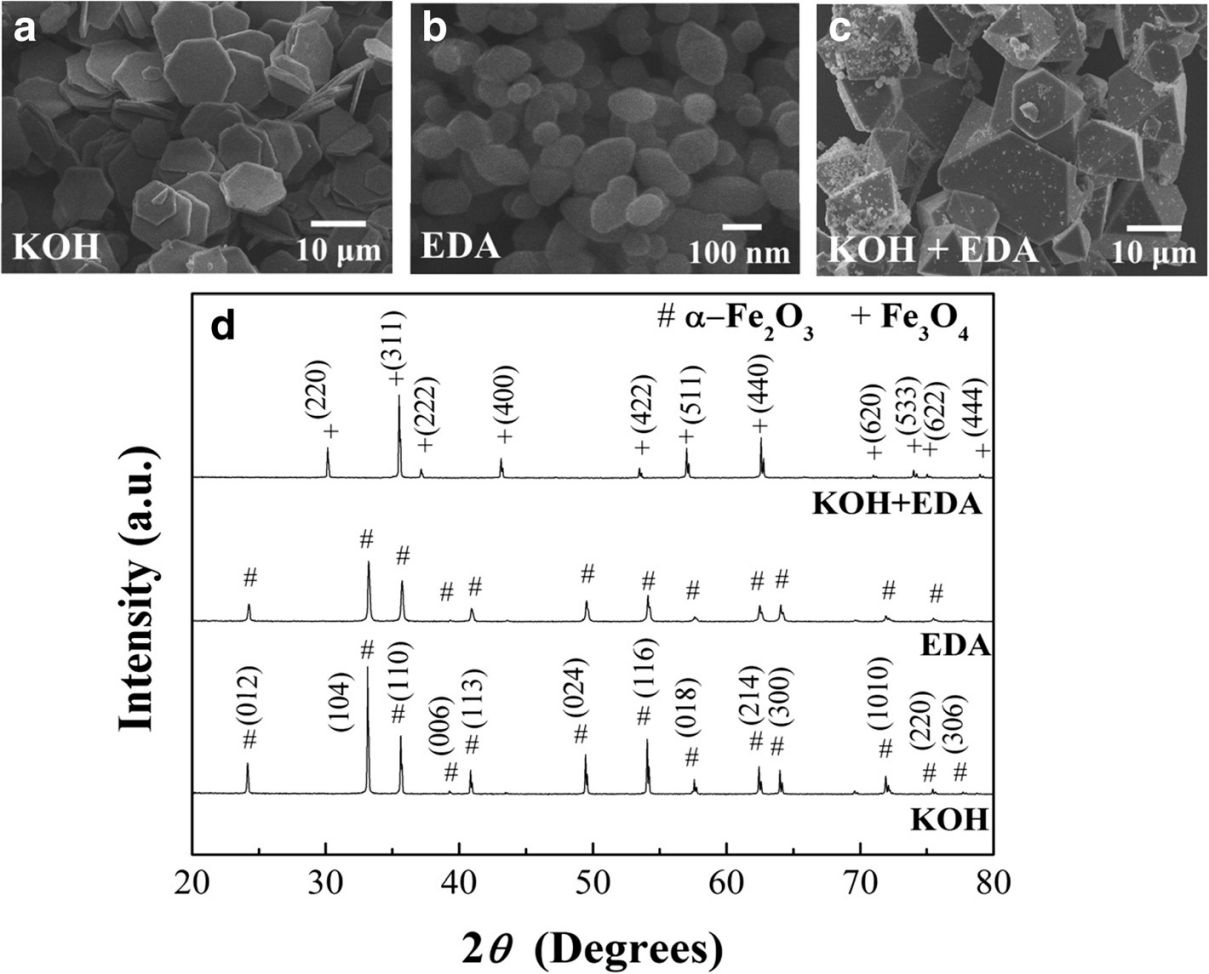
**SEM images and corresponding XRD patterns of iron oxide particles.** SEM images of iron oxide particles prepared with the addition of **(a)** 5 ml of 10.67 M KOH, **(b)** 1 ml of EDA, and **(c)** both 5 ml of 10.67 M KOH and 1 ml of EDA into the ferric solutions. **(d)** The corresponding XRD patterns of the iron oxide particles obtained for the cases of **(a)**, **(b)**, and **(c)**.

**Figure 2:**
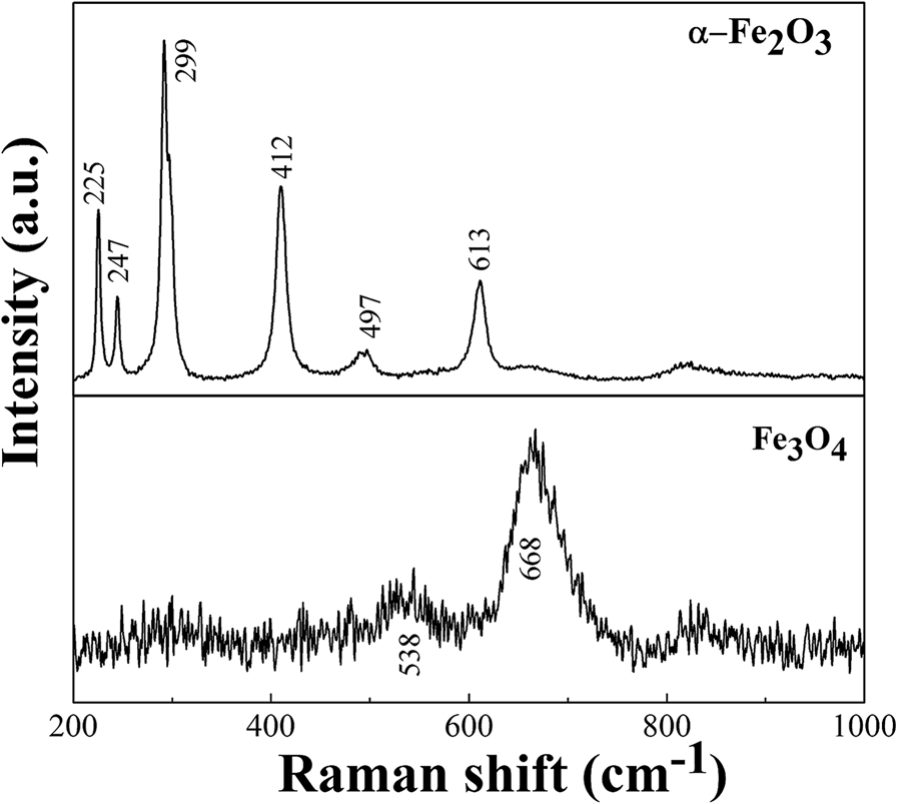
**Raman spectra of α-Fe**_**2**_**O**_**3 **_**hexagonal plates and Fe**_**3**_**O**_**4 **_**polyhedral particles.**

Figure 1 shows the iron oxide particles synthesized with three different reducing agents, KOH, EDA, and KOH/EDA, under a hydrothermal condition of 200°C for 9 h in the ferric solution. Figure 1a shows the α-Fe_2_O_3_ hexagonal plates which were obtained with the addition of KOH, and Figure 1b shows the α-Fe_2_O_3_ hexagonal bipyramid particles obtained when EDA was added into the system. Figure 1c shows the Fe_3_O_4_ polyhedral particles obtained with the addition of both KOH and EDA into the reaction system. (When NaOH substitutes for KOH, a similar reaction would occur.) The crystal structure of these iron oxide particles was analyzed by XRD and is shown in Figure 1d. The phase can be identified to be α-Fe_2_O_3_ when either KOH or EDA alone was added to the reaction system despite different morphologies. The diffraction peaks match the JCPDS card no. 33-0664 which is a rhombohedral structure with space group $R\bar{3}c$. The diffraction peaks obtained with the addition of both KOH and EDA into the reaction system correspond to the phase of Fe_3_O_4_, JCPDS card no. 19-0629, which is a face-centered cubic structure with space group $\mathrm{Fd}\bar{3}m$. The characteristic reflections in the Fe_3_O_4_ phase and the γ-Fe_2_O_3_ phase are about the same [38]. Here diffraction of the (221), (210), and (213) planes for the γ-Fe_2_O_3_ phase does not exist. To further clarify the phase of polyhedral particles, the Raman spectra of α-Fe_2_O_3_ hexagonal plates and Fe_3_O_4_ polyhedral particles are shown in Figure 2. α-Fe_2_O_3_ here can be characterized by four strong peaks at around 225, 299, 412, and 613 cm^-1^ and two weak peaks around 247 and 497 cm^-1^. The peaks at 538 and 668 cm^-1^ were attributed to Fe_3_O_4_, while the peaks at 350, 500, and 700 cm^-1^ belonging to γ-Fe_2_O_3_ were not observed [39,40]. The appearance of the Fe_3_O_4_ phase during reaction is a clear evidence that the valence change from Fe^3+^ to Fe^2+^ must occur due to the fact that Fe^2+^ ions occupy the octahedral sites of Fe_3_O_4_.

The α-Fe_2_O_3_ hexagonal plates have an average size of about 10 μm in edge length and about 500 nm in thickness. The average lateral size of the α-Fe_2_O_3_ particles with the shape of a hexagonal bipyramid is about 120 nm. The Fe_3_O_4_ polyhedral particles with mainly octahedral shape have an average lateral size in the range of 5 to 25 μm. The particles obtained from the reaction system with the addition of KOH and EDA alone have the same phase but different shapes. One would assume that the reaction system with the addition of both KOH and EDA would produce particles with maybe different shapes but still maintain the phase of α-Fe_2_O_3_. However, the results show that the particles that we obtained have a different phase, Fe_3_O_4_, and, surely, a different shape.

**Figure 3:**
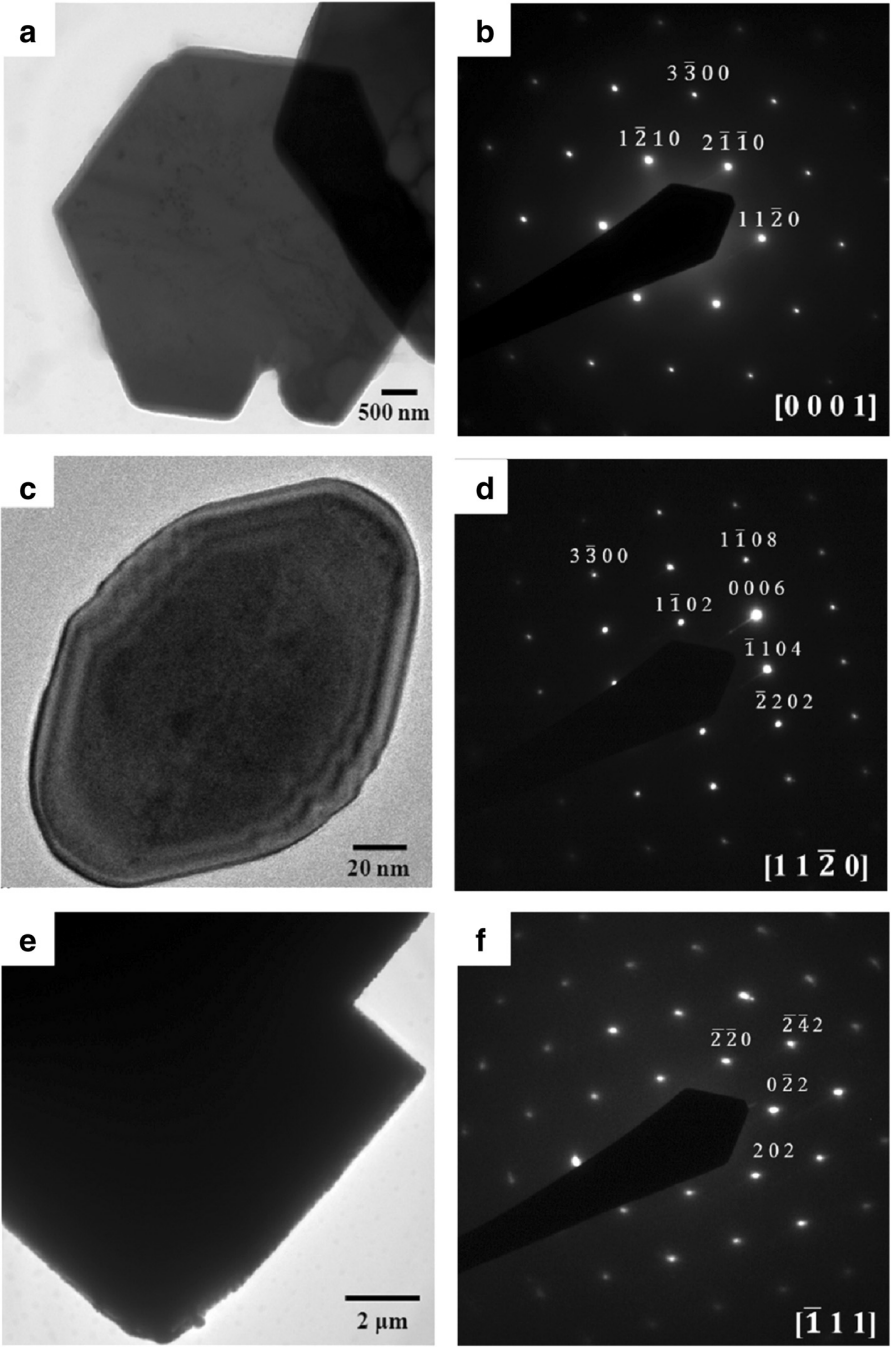
**TEM images and SAED patterns of α-Fe**_**2**_**O**_**3 **_**hexagonal plates (a, b), α-Fe**_**2**_**O**_**3 **_**hexagonal bipyramid (c, d), and Fe**_**3**_**O**_**4 **_**polyhedral particles (e, f).**

The transmission electron microscopy images and the corresponding selected area electron diffraction (SAED) patterns of iron oxide particles are shown in Figure 3. The diffraction patterns of the particles confirmed the results of the XRD diffractions. In Figure 3b, the zone axis of the hexagonal plate is [0001] and the six directions normal to the edge are $<2\bar{1}\bar{1}0>$ and its other five equivalent directions. In Figure 3d, the hexagonal bipyramid shows that the pyramid is pointed in the direction of <0001>. According to the literatures, the bipyramidal structure was enclosed by $\left\{10\bar{1}1\right\}$ crystal planes [41]. In Figure 3f, the Fe_3_O_4_ polyhedral particles which are composed of the pure magnetite phase and the diffraction spots are identified to be (202), $\left(0\bar{2}2\right)$, and $\left(\bar{2}\bar{2}0\right)$ planes and their equivalent planes under an incident electron beam along $\left[\bar{1}11\right]$.

**Figure 4:**
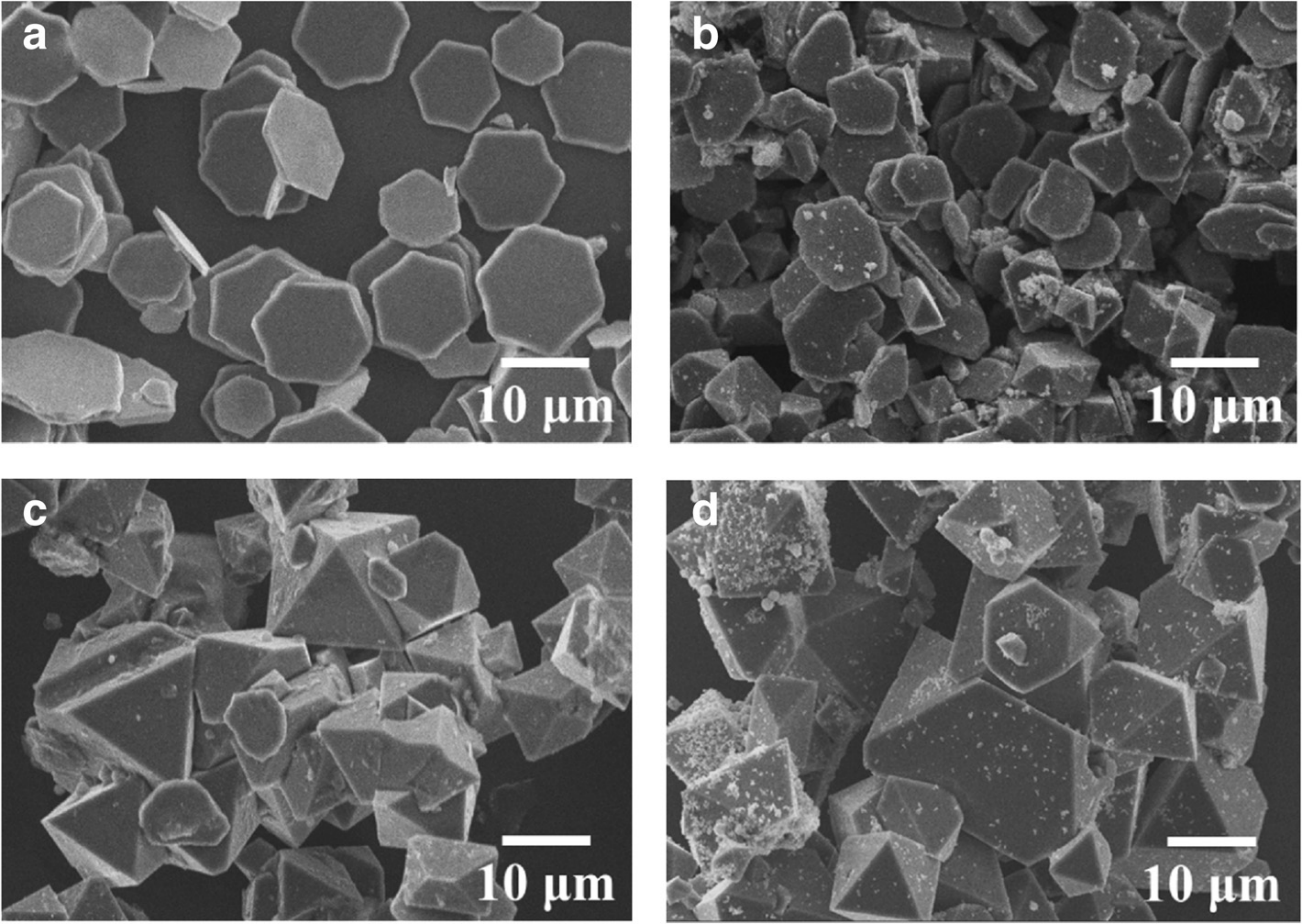
**Mixture of α-Fe**_**2**_**O**_**3 **_**and Fe**_**3**_**O**_**4 **_**particles precipitated in the hydrothermal system at 200 °C at different times. (a)** 2 h, **(b)** 5 h, **(c)** 7 h, and **(d)** 9 h.

To further understand the formation process of Fe_3_O_4_, the reaction systems with the addition of both KOH and EDA were hydrothermally synthesized at 200°C for different reaction times, as shown in Figure 4. Figure 4a shows that, after 2 h of growth, the main phase of the particles is α-Fe_2_O_3_ hexagonal plates. The edge of the hexagonal plate is not as straight as that obtained for the reaction system with KOH only. As the reaction time increased to 5 h, as shown in Figure 4b, small octahedron particles were observed and the original hexagonal plate started to dissolve and no longer maintained the hexagonal shape. As the reaction time continued to increase to 7 h, more polyhedron particles were observed with larger sizes and only a small amount of plate-like particles still existed, as shown in Figure 4c. At the reaction time of 9 h, the observed particles are mainly polyhedron ones, as shown in Figure 4d. The first observation in this sequence of experiment is that KOH can rapidly transform iron hydroxides to hematite. The second observed phenomenon is that the α-Fe_2_O_3_ hexagonal plates were dissolved to become irregular plates during the transformation process.

The result implied that phase transformation evolved in four steps: (1) the reaction systems rapidly transformed Fe(OH)_3_ or FeOOH to α-Fe_2_O_3_ hexagonal plates under the hydrothermal conditions, (2) the α-Fe_2_O_3_ hexagonal plates dissolved gradually, (3) the reduction process causes valence transition of Fe^3+^ to Fe^2+^, and (4) the Fe_3_O_4_ particles started to nucleate and then finally grew to form polyhedral particles.

**Figure 5:**
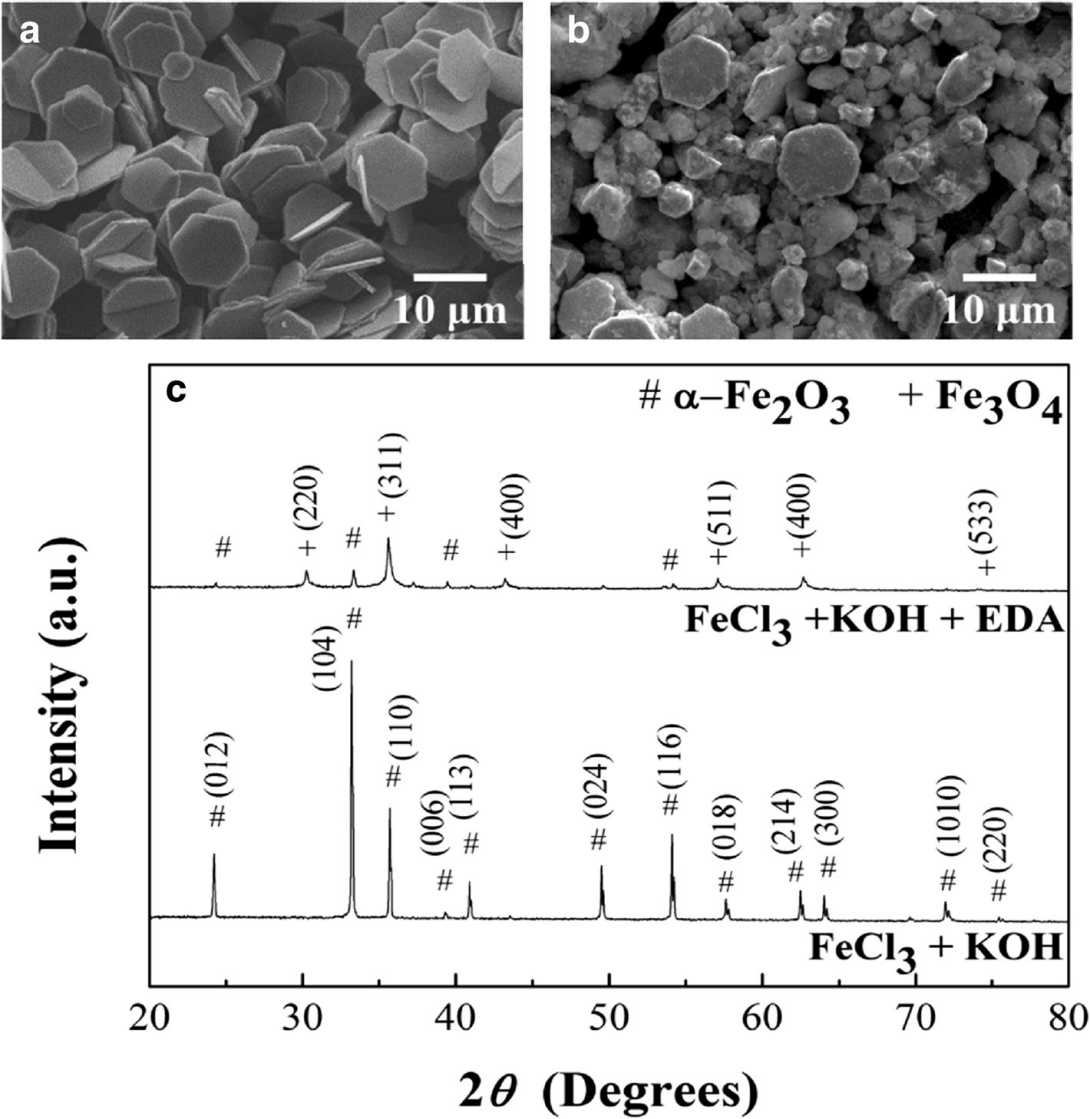
**SEM images and corresponding XRD patterns of iron oxide particles.** SEM images of iron oxide particles formed with **(a)** FeCl_3_ + KOH and **(b)** FeCl_3_ + KOH + EDA. **(c)** The corresponding XRD patterns of iron oxide obtained for the cases of **(a)** and **(b)**.

To further understand the role of NO_3_^-^ ions on the phase transition process, the precursor of FeNO_3_ was substituted by FeCl_3_ with the same hydrothermal conditions. Two cases were investigated, one with the addition of KOH only and the other with the addition of both KOH and EDA under the same hydrothermal condition of 200°C for 9 h. Figure 5a shows that the α-Fe_2_O_3_ hexagonal plates were obtained when the reaction system consists of FeCl_3_ and KOH, while the phase transformation from α-Fe_2_O_3_ hexagonal plates to Fe_3_O_4_ polyhedral particles still occurred when the reaction system consists of FeCl_3_, KOH, and EDA, as shown in Figure 5b. The shape of the polyhedral particles is more irregular in this case. The XRD patterns, shown in Figure 4c, confirmed the related phases. Notice that the α-Fe_2_O_3_ plates were not completely reduced to Fe_3_O_4_ particles. Thus, NO_3_^-^ ions are not directly involved in the reduction process of Fe^3+^ to Fe^2+^. However, the transformation process is faster with the presence of NO_3_^-^ ions in the reaction system than that of Cl^-^ ions.

**Figure 6:**
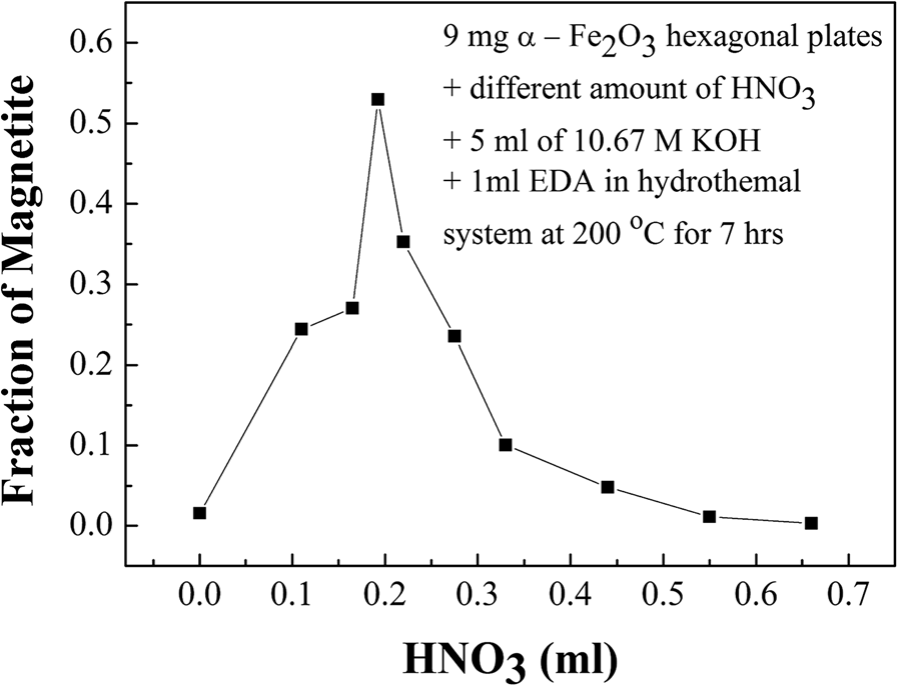
**The fraction of magnetite transformed with different amounts of HNO**_**3**_**.** HNO_3_ was added to 9 mg of pre-synthesized α-Fe_2_O_3_, 5 ml of 10.67 M KOH, and 1 ml of EDA under hydrothermal process at 200°C for 7 h.

We further explore the role that NO_3_^-^ ions play on the phase transition. The pre-synthesized α-Fe_2_O_3_ hexagonal plates of 9 mg were added to the same KOH and EDA medium as above but with different amounts of HNO_3_ and heated to 200°C for 7 h. As shown in Figure 6, the results show that the phase transition rates were slow when the solution contained large and small amounts of HNO_3_; the optimal amount of HNO_3_ for phase transition is 0.19 ml. The slow phase transition rate observed for small amount of HNO_3_ may be attributed to the limiting dissolution of α-Fe_2_O_3_ which produced Fe^3+^ ion in the solution for further reduction to Fe^2+^. Thus, the rate of phase transformation is slow. At large amount of HNO_3_, the NO_3_^-^ ions can be the oxidant in the reaction [29] and the pH value of the reaction system is changed toward a less basic solution. Hence, the reduction process can be again suppressed. Thus, there is a proper amount of HNO_3_ that induces the maximum rate for phase transformation.

A similar *in situ* reduction capability of EDA in neutral and basic solutions for the reduction of uranium from U^6+^ to U^4+^ has been reported by Jouffret et al. [42]. In our study, the phase transition process should be similar. The EDA maintains stable and chelates with Fe^3+^ ions that were released by α-Fe_2_O_3_ hexagonal plates upon dissolving, and the reduction of Fe^3+^ ions to Fe^2+^ ions occurred.

**Figure 7:**
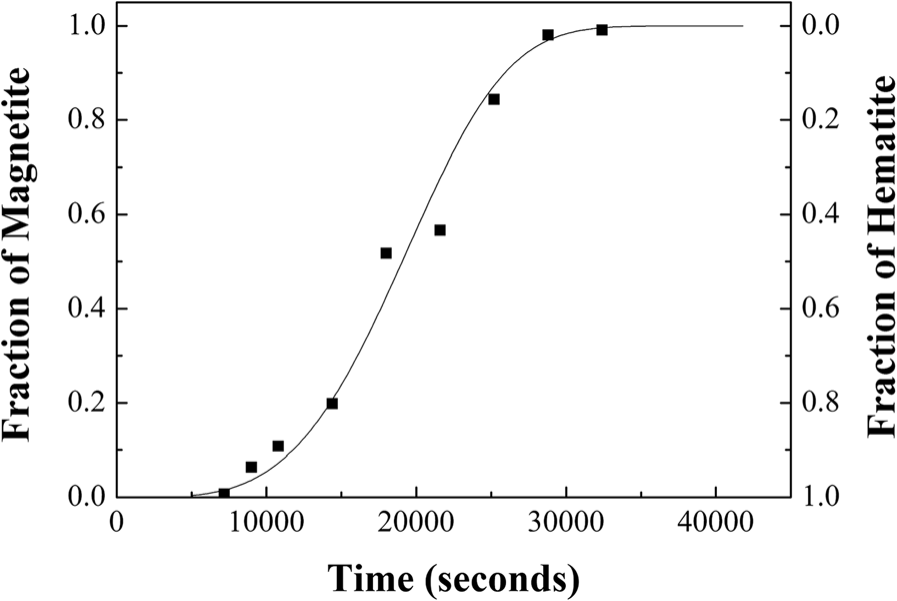
**The fraction of magnetite transformed as a function of reaction time for Fe(NO**_**3**_**)**_**3**_**, KOH, and EDA.** Under hydrothermal reaction at 200°C.

Figure 7 shows the curve of transformed fraction of magnetite (*α*) as a function of reaction time. The fraction of α-Fe_2_O_3_ and Fe_3_O_4_ was determined by XRD measurement in conjunction with the Rietveld method. By using the Avrami equation, *α* = 1 - exp(-*kt*^*n*^), where *k* is the reaction constant, *t* is the reaction time, and *n* is the exponent of reaction, we can fit, relatively well, the experiment data of the magnetite fraction obtained by hydrothermal treatment at 200°C for different times. The value of *n* is about 4 obtained in this case. From this curve, we can further investigate the kinetic behavior of phase transformation in the reaction condition in the future.

**Figure 8:**
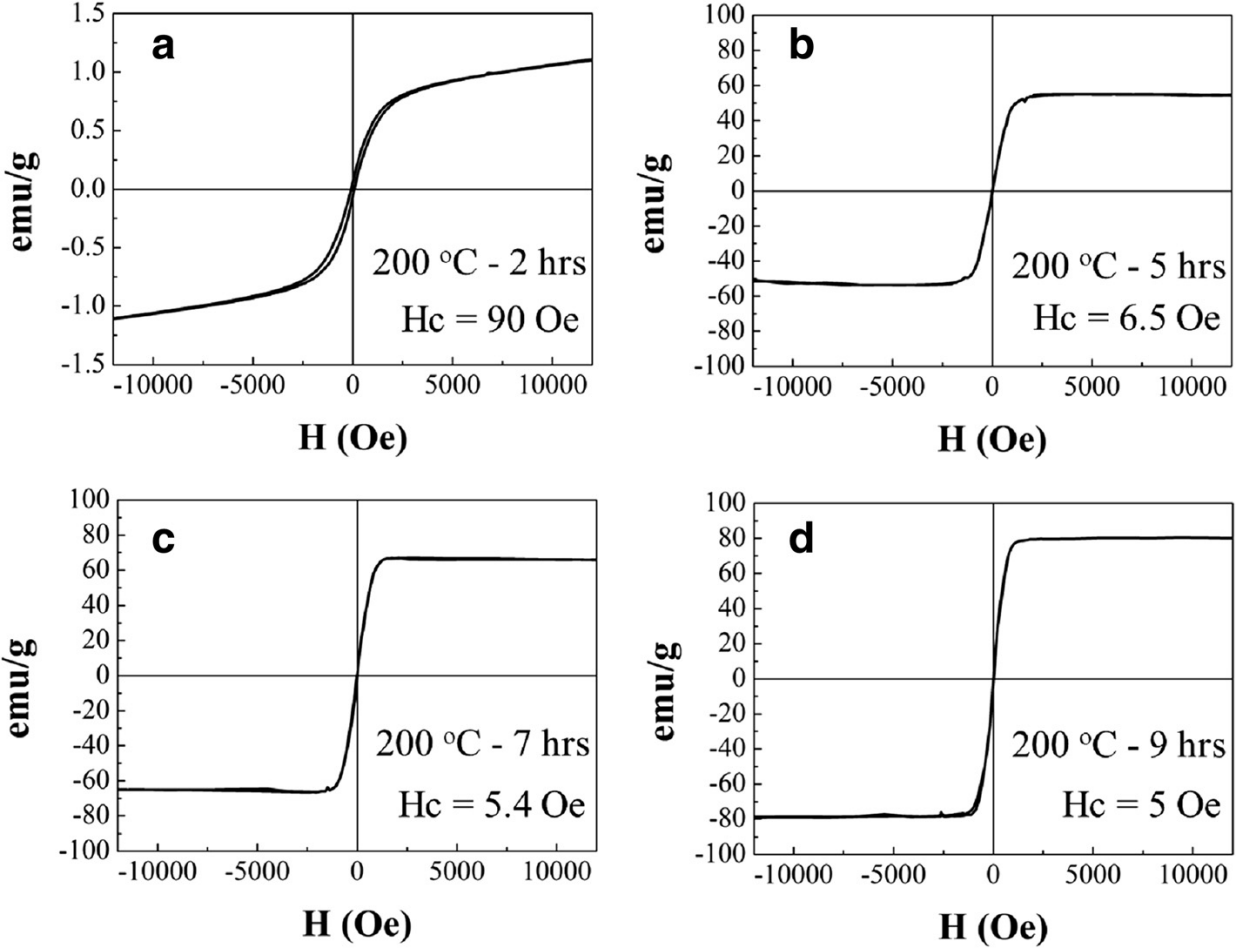
**Magnetic properties of mixed α-Fe**_**2**_**O**_**3 **_**and Fe**_**3**_**O**_**4 **_**particles prepared by hydrothermally induced phase transformation at 200°C. (a)** 2 h, **(b)** 5 h, **(c)** 7 h, and **(d)** 9 h.

The magnetic properties of iron oxide particles followed the phase transition process from α-Fe_2_O_3_ hexagonal plates to Fe_3_O_4_ polyhedral particles, as shown in Figure 8. After a short reaction time of 2 h, the α-Fe_2_O_3_ hexagonal plates show weak ferromagnetic behaviors with a coercive force of 90 Oe at room temperature and the saturation magnetization is yet to reach the maximum in the range of the applied magnetic field, as shown in Figure 7a. Prolonging the reaction time to 5 ~ 7 h, the fraction of Fe_3_O_4_ polyhedral particles as well as the particle size of Fe_3_O_4_ increases gradually. As shown in Figure 7b,c, the values of saturation magnetization increase to 55 and 66 emu/g and the coercive forces decrease to 6.5 and 5.4 Oe for the reaction time of 5 and 7 h, respectively. Finally, the phase transition was completed at the reaction time of 9 h. The Fe_3_O_4_ polyhedral particles show strong ferromagnetic behaviors with the highest saturation magnetization of 80 emu/g and the lowest coercive force of 5 Oe, as shown in Figure 7d. The magnetic properties of α-Fe_2_O_3_ hexagonal plates and Fe_3_O_4_ polyhedral particles are similar to the previous reports [27,43].

## Conclusions

α-Fe_2_O_3_ nano/microhexagonal plates can be successfully reduced to octahedral Fe_3_O_4_ particles with EDA in an alkaline solution under a low-temperature hydrothermal process. In general, the transformation consists of four stages: (1) the formation of α-Fe_2_O_3_ hexagonal plates triggered by KOH, (2) the dissolution of the α-Fe_2_O_3_ hexagonal plates, (3) the reduction of Fe^3+^ to Fe^2+^, and (4) the nucleation and growth of new Fe_3_O_4_ polyhedral particles. The Avrami equation can be used to describe the transformation kinetics. As the phase transformation proceeded, the magnetic properties of the sample gradually transformed from weak ferromagnetic behaviors to strong ferromagnetic behaviors.

## Competing interests

The authors declare that they have no competing interests.

## Authors’ contributions

JFL wrote the manuscript and performed all the experiments and the data analysis. CJT provided the information and organized the final version of the paper. Both authors read and approved the final manuscript.

## Authors’ information

JFL is a Ph.D. student at National Tsing Hua University. CJT holds a professor position at National Tsing Hua University.
